# Combinations of long-term care insurance services and associated factors in Japan: a classification tree model

**DOI:** 10.1186/1472-6963-14-382

**Published:** 2014-09-10

**Authors:** Ayumi Igarashi, Tomoaki Ishibashi, Tomohiro Shinozaki, Noriko Yamamoto-Mitani

**Affiliations:** School of Health Sciences & Nursing, Graduate School of Medicine, The University of Tokyo, 7-3-1 Hongo, Bunkyo-ku, Tokyo, 113-0033 Japan; The Dia Foundation for Research on Ageing Societies, 1-34-5 Shinjuku-ichome, Shinjuku-ku, Tokyo, 160-0022 Japan

**Keywords:** Service utilization, Care management, Home care, Older adult, Chi-squared automatic interaction detection

## Abstract

**Background:**

To develop a quality community-based care management system, it is important to identify the actual use of long-term care insurance (LTCI) services and the most frequent combinations of services. It is also important to determine the factors associated with the use of such combinations.

**Methods:**

This study was conducted in 10 care management agencies in the urban area around Tokyo, Japan. The assessment and services data of 983 clients using the Minimum Data Set for Home Care were collected from the agencies. We categorized combination patterns of services from descriptive data analysis of service use and conducted chi-squared automatic interaction detection (CHAID) analysis to identify the primary variables determining the combinations of the services used.

**Results:**

We identified nine patterns of service use: day care only (16.5%); day care and assistive devices (14.4%); day care, home helper, and assistive devices (13.2%); home helper and assistive devices (11.8%); assistive devices only (10.9%); home helper only (8.7%); day care and home helper (7.7%); home helper, visiting nurse, and assistive devices (5.4%); and others (11.3%). The CHAID dendrogram illustrated the relative importance of significant independent variables in determining combination use; the most important variables in predicting combination use were certified care need level, living arrangements, cognitive function, and need for medical procedures. The characteristics of care managers and agencies were not associated with the combinations.

**Conclusion:**

This study clarified patterns of community-based service use in the LTCI system in Japan. The combinations of services were more related to the physical and psychosocial status of older adults than to the characteristics of agencies and care managers. Although we found no association between service use and the characteristics of agencies and care managers, further examination of possible bias in the use of services should be included in future studies. Researchers and policymakers can use these combinations identified in this study to categorize the use of community-based care service and measure the outcomes of care interventions.

## Background

The rapidly aging society in Japan led to the introduction of mandatory public long-term care insurance (LTCI) in 2000. The goal of the LTCI was to develop a system of social care that would help older people live independently regardless of disability [1, 2]. It covers long-term nursing care and social services for persons aged 65 years and over, as well as those between 40 and 64 years with specific age-related diseases [3]. In the LTCI, eligibility of care is determined at two support levels and five care need levels; each level has a ceiling on the amount of services that can be used, ranging from $400 to $2900 per month [4]. Following the introduction of the LTCI, the supply of formal community-based care services has increased rapidly, and the number of older people who use these services has also increased, from 971,461 in June 2000 to 3,420,700 in April 2013 [5]. Because of the increasing use of services, providing quality services has become even more important.

Under the LTCI, the necessary services for each older adult are determined by a comprehensive assessment by a care manager (CM), a new profession introduced by the LTCI. CMs come from a variety of professional backgrounds, including care workers, social workers, and nurses. CMs work in care management agencies that are operated by several types of organizations, such as for-profit, social welfare, and non-profit organizations as well as healthcare corporations. Many of these organizations operate more than one agency and provide other care services (e.g., home helper, visiting nurse, day care, and assistive devices). Developing a high-quality care management system in the LTCI requires knowledge of how services are provided under the management of CMs.

To identify how the long-term care services are used, it is necessary to determine the factors associated with the use of services. Many studies have examined factors associated with the use of the following services: home helper [6–11], day care [12], and visiting nurse [2, 9, 10, 13], nutrition/meals-on-wheels services [8, 10], and any combination of two or more long-term care services [8, 14]. In other studies, researchers have examined the rate of utilization for each service [15] and the cost of services [10, 16]. In most of these studies, the independent variables associated with the use of services were selected based on the Anderson-Newman model of predisposing, enabling, and need factors [17]. Predisposing factors associated with long-term care service use included age [8, 11] and gender [8], while enabling factors associated with use of services included living arrangement [8, 10, 11, 16], social support [8], informal care [10], and caregiver’s burden [7, 18]. Among these factors, the familial relationship of the primary caregiver was also identified as an important determinant of service use. Spousal caregivers were more likely to receive outside assistance than adult children caregivers [19]. Need factors associated with the use of services included functional disability [7–9, 11]; cognitive decline or disability [18]; specific diseases like stroke, heart, and respiratory diseases [8, 16]; and the perceived need for services [9, 10].

In addition, it has been suggested that service selections are affected by the management skills of the CMs; the variety of professional backgrounds and the potential conflict of interest in recommending services that are offered by the service agency that employs the CM may affect combinations of services [20]. Although Kashiwagi et al. [2] demonstrated an effect of the type of care management corporation on the use of visiting nurses—the service was provided more frequently for clients managed by a medical care management agency than clients served by a non-medical care management agency—no study has confirmed the effect of other characteristics of care management agencies (e.g., whether they provide other services, such as home helper and day care services, operated by the same organization), CMs, and the effect of these characteristics on the use of other types of services.

It is also important to consider the combinations of services used in addition to the factors related to service use. Although the Japanese government reports usage statistics of each care service in the LTCI system every month on its website [21], how the services are combined to meet the needs of individuals is not reported. To analyze the combinations of services, some studies have used pre-determined “service packages”; that is, set combinations of services. Such combinations have consisted of in-home (visiting), out-of-home (ambulatory), and mixed services [22, 23] or visiting, ambulatory, short-stay respite care (including in a hospital or a long-term care facility), and institutional services [24]. These studies, however, decided service combinations in an arbitrary manner without consideration of actual use.

In contrast, researchers in other countries have categorized patterns of long-term care service use in empirical analyses [25–27]. For example, Kendig et al. classified home/community care service use into nine patterns using cluster analysis and enumerated the characteristics of each pattern [26]. These previous studies, however, classified tendencies of service combinations into patterns; various combinations of services were involved in each pattern, and unified or fixed combinations were not determined.

We found only one study that identified the combinations of services based on actual combinations and examined factors associated with the service use [28]. However, in that study, the combinations did not include the use of assistive devices and day care, both of which are commonly used LTCI services in Japan. Further, in the analysis, multinomial logistic regression was used with visiting nurse service as a reference; the differences between types of the combinations of services other than the nursing service could not be examined.

For a more complete evaluation of services and the development of strategies to improve the quality of care management, it is important to identify the actual use of LTCI services, including combinations of services, and to examine factors associated with these combinations, which are likely to include the characteristics of older adult clients, care management agencies, and CMs. Thus, this study sought to identify the patterns of combined community-based LTCI services used by older adults in need of care and the factors related to the selection of these service combinations.

## Methods

### Study design and participants

This study was part of a quality evaluation and improvement project in community-based care management using Home Care Quality Indicators based on the Minimum Data Set-Home Care (MDS-HC) [29]. The MDS-HC is a comprehensive assessment tool [30] for older adults who have been officially designated for assessment and care planning by CMs in the Japanese LTCI system. For the quality evaluation and improvement project, we recruited care management corporations that used the MDS-HC in 2010. Four corporations with 10 care management agencies, all located in the Greater Tokyo Area, agreed to participate in the project. One corporation was a healthcare facility and the other three were for-profit; all corporations had agencies for assistive devices. At the agency level, all agencies supplied home helper services; two agencies (the healthcare facility and one of the for-profit corporations) supplied visiting nurse services; one agency supplied day care services; and one agency had bathing services. The participating corporations introduced a cloud-based assessment system customized for this study, which enabled us to download assessment and services data.

Although the participating agencies conducted care management for older adults at all care need and support levels, we decided to include only clients assigned a care need level in this study for two reasons: there were very few clients in the levels of support in the assessment databases and, in most cases, individuals in these levels used just one service because of their limited benefits [31]. We retrieved assessment data for all clients with a care need level who had been assessed between November 2010 and October 2011, along with their care plan data, and identified the use of LTCI services three months after the assessment. When there was more than one assessment per client during this period, we selected the most recent assessment. This resulted in assessment data for 1160 individuals. We then matched the assessment and care plan data for each client. If the LTCI-certified care need level changed during the three months following the assessment, we excluded the client data from analyses; we eventually included the data of 983 clients in the analyses. To investigate CM characteristics, we conducted a self-reported questionnaire survey targeting CMs in May 2012. The data of each CM was then matched to the client data.

To maintain client and CM anonymity, a data download system was developed that automatically changed the client and CM identification numbers. Although the identification numbers were linked, the researchers were not allowed to connect the data. The Ethics Committee of the Dia Foundation for Research on Ageing Societies approved this study.

### Measures

The data used in this study included the use of LTCI services and the characteristics of older adults, care management agencies, and care managers.

#### Services utilized in long-term care insurance

We investigated the use of LTCI services by each client according to their care plan. The community-based services in the LTCI system consist of home helper, day care, day care with rehabilitation, visiting nurse, assistive devices, bathing, home rehabilitation, short-stay respite care, and management guidance for in-home care [1, 4, 32]. In this study, we combined the use of day care and day care with rehabilitation as “day care” because they play a similar role of respite care for families during the day and rehabilitation in the LTCI, and the rate of the use of day care with rehabilitation was relatively low. Further, we excluded the utilization of management guidance for in-home care from the analyses because it was not available from the care plan data in the care management agencies.

#### Characteristics of clients, care management agencies, and care managers

The Andersen-Newman model [17] was used to select independent variables regarding the characteristics of clients, care management agencies, and CMs that could be associated with the service use. We defined gender and age of clients as predisposing factors; socioeconomic status of clients such as living arrangement, caregiver’s situation and economic status, the characteristics of agencies, and CMs as enabling factors; care need level, diagnoses, and physical and psychological status of clients as need factors.

The characteristics of clients were assessed using MDS-HC assessment, including demographics and physical, psychological, and socioeconomic status. The demographics included age, gender, LTCI-certified care need level (ranging from levels 1 to 5), and diagnoses (cerebrovascular disease, dementia, diabetes, arthritis, fracture, cardiac disease, and cancer). The client’s living arrangement (living with family or alone) and relationship to the primary caregiver were also included, as well as the economic status of the client.

The physical status of clients was assessed on activities of daily living (ADL), the presence or absence of pain, difficulty swallowing, urinary incontinence, fecal incontinence, a fall in the past 90 days, pressure ulcers, and the need for any medical procedures (oxygen administration, vascular infusion, and catheter or fistula). ADL were measured using the ADL Hierarchy Scale, which uses information on self-performance of ADL, such as bathing and eating, to assign scores from 0 (*no impairment*) to 6 (*total dependence on caregiver);* this scale has adequate validity [33]. Pain was measured on a four-point Pain Scale that also has adequate validity [34]; pain scores ranged from 0 (*no pain*) to 3 (*severe pain*). Dichotomous scores (0, 1) were used to assess whether clients had difficulty swallowing, urinary incontinence, fecal incontinence, falls, and pressure ulcers.

Measures of cognitive function and depression were used to assess clients’ psychological status. Cognitive function was measured with the Cognitive Performance Scale (CPS), which uses the MDS-HC data on memory and communication skills to assign clients a score from 1 to 7; scores ≥3 are indicative of moderate to severe cognitive impairment, and this scale has adequate validity [35]. Depression was measured with the Depression Rating Scale (DRS), which consists of seven items to create a 14-point scale in which scores ≥3 serve as a marker for depressive symptoms; this scale has adequate reliability and validity [36]. All scale scores were available from the MDS-HC data.

Agency characteristics included the agency’s code (identification number), and the corporation code and type (such as for-profit or healthcare facility) of the organization that manages the care management agency and affiliated service agencies providing care (visiting nurse, day care, bathing, and healthcare facilities).

The characteristics of CMs that were obtained from the questionnaire completed by the CMs in charge of the clients included age, gender, professional background (health care professional, such as nurse; or non-health-care professional, such as a care worker or social worker), and years of experience, both in the background profession and working as a CM.

### Statistical analyses

First, we conducted descriptive analyses of the characteristics of the CMs, clients, and services (Table 1). Second, we classified the combinations of services into categories from the descriptive data of each service. We focused on several types of services and examined the associations between the identified service combinations and remaining minor services (Table 2).

**Table 1:** **Characteristics of care managers, clients, and services**

		n	%	Tokyo %
**Characteristics of care managers (n = 48)**				
Age (years)	20–29	1	2.1	
	30–39	13	27.7	
	40–49	10	21.3	
	50–59	19	40.4	
	60 and over	4	8.5	
Gender	Female	37	90.2	
Long-term care work experience facility/hospital	None	17	37.8	
	Facility	13	28.9	
	Hospital	15	33.3	
Professional background	Healthcare	7	15.2	
	Non-healthcare	39	84.8	
Years of experience in profession, mean ± SD		12.0 ± 4.9		
Years of working as care manager, mean ± SD		4.9 ± 3.0		
**Characteristics of Clients (n = 983)**				
Age (years), mean ± SD		80.0 ± 9.9		
Gender	Female	622	64.1	
Care need level	1	280	28.5	29.3
	2	278	28.3	30.1
	3	163	16.6	18.0
	4	137	13.9	12.8
	5	125	12.7	9.8
Diagnosis				
Cerebrovascular disease		298	30.3	
Dementia		271	27.6	
Diabetes		159	16.2	
Arthritis		118	12.0	
Fracture		133	13.5	
Cardiac disease		131	13.3	
Cancer		71	7.2	
Living with family		700	79.5	
Relationship with caregiver				
Child or his/her spouse		538	58.8	
Spouse		308	33.7	
Other		69	7.5	
Economic status	Poverty	34	3.7	
Physical status				
ADL^a^ mean ± SD		1.6 ± 1.8		
Independent		430	43.7	
Supervision		166	16.9	
Limited		111	11.3	
Extensive		77	7.8	
Maximal		87	8.9	
Dependent		84	8.5	
Total Dependent		28	2.8	
Difficulty swallowing		211	22.0	
Urinary incontinence				
Once or less/week		680	70.5	
Twice or more/week		284	29.5	
Fecal incontinence				
None		768	80.0	
Once or more/week		192	20.0	
Pain^b^				
No pain		487	51.5	
Mild pain		204	21.6	
Moderate pain		208	22.0	
Excruciating pain		47	5.0	
Fall (past 90 days)				
None		723	79.2	
Once or more		190	20.8	
Pressure ulcer		41	4.3	
Psychological status				
Cognitive function^c^				
Intact to mild disabilities		626	64.6	
Moderate to severe disabilities		343	35.4	
Depression^d^		93	9.5	
**Service use in home care**				
Assistive devices		630	64.1	60.9
Day care		570	58.0	60.3
Home helper		512	52.1	52.2
Visiting nurse		160	16.3	18.7
Short-stay respite care		156	15.9	11.6
Bathing		114	11.6	6.0
Home rehabilitation		24	2.4	3.2

SD: standard deviation.

^a^ADL was measured on the ADL Hierarchy Scale.

^b^Pain was measured on a pain scale.

^c^Cognitive function was measured on Cognitive Performance Scale (CPS).

^d^Depression was measured on Depression Rating Scale (DRS).

**Table 2:** **Patterns of service use (n = 983)**

	D		D & A		D, H, & A		H & A		A		H		D & H		H, N, & A		Other	
	n = 162		n = 142		n = 130		n = 116		n = 107		n = 86		n = 76		n = 53		n = 111	
	n	%	n	%	n	%	n	%	n	%	n	%	n	%	n	%	n	%
Short-stay respite care	**30**	**18.5**	**41**	**28.9**	**26**	**20.0**	10	8.6	4	3.7	2	2.3	8	10.5	7	13.2	**28**	**25.2**
Bathing	2	1.2	4	2.8	3	2.3	**28**	**24.1**	17	15.9	2	2.3	0	0.0	**31**	**58.5**	**27**	**24.3**
Home Rehabilitation	1	0.6	0	0.0	2	1.5	6	5.2	6	5.6	2	2.3	1	1.3	4	7.5	2	1.8

D: day care, H: home helper, A: assistive devices, N: visiting nurse.

Bold numbers represent frequent patterns of minor services used.

Finally, to identify the significant variables that affected the combinations of services used, we conducted chi-squared automatic interaction detection (CHAID) analysis [37], commonly used in the field of data mining. The CHAID technique determines the relative importance of each of the independent (predictor) variables in explaining group membership in a categorical dependent (outcome) variable with *χ*^2^ significance level. We used the combinations of services as the dependent variable and the characteristics of clients, agencies, and care managers as independent variables.

According to a previous study using the technique [38, 39], we selected the following criteria to prevent inappropriate splitting of nodes: the tree depth was limited to three levels, no group smaller than 40 was split (parent node), and no group smaller than 20 was formed (child node). To confirm the validity of the prediction accuracy (i.e., the correct classifications) of the models developed using the CHAID algorithm, we ran a “cross-validation command”: the sample was divided randomly into 10 groups (a default of the program), and a dendrogram was created using nine subsamples. Next, the dendrogram was applied to the remaining one subsample for the test and the proportion of correct classifications was calculated. This examination was repeated nine more times with the subsample for the test changed each time. The average proportion across the 10 test subsamples is discussed in the following section. We used the dendrogram that was drawn using the full sample as the final model.

Further, to deal with the possibility of differences in the use of day care and day care with rehabilitation, we conducted analyses with these as separate categories.

SPSS, version 21, and Decision Tree, version 21 (IBM, Chicago, IL, USA), were used for the analyses. The significance level was set at less than 0.05 (two-tailed).

## Results

### Characteristics of care managers, clients, and services

Table 1 shows the characteristics of CMs, clients, and services used by clients. The questionnaire was not completed by the CMs of one corporation because those CMs had a high amount of daily routine work.

Ninety percent of the 48 CMs were female and 15% had a professional healthcare background (e.g., nurse); their years of experience in their profession and as CMs were 12.0 and 4.9, respectively. The mean age ± standard deviation (SD) of the clients was 80.0 ± 9.9; 64% were female. The distribution of care need level in this study was comparable with that of the population in Tokyo, with only a slightly larger rate of higher care need levels [40]. About 40% of the clients were ADL independent and cognitively intact.

The proportion of the service utilizations were as follows: assistive devices, 64.1%; day care, 58.0%; home helper, 52.1%; visiting nurse, 16.3%; short-stay respite care, 15.9%; bathing, 11.6%; and home rehabilitation, 2.4%. These rates were also comparable to population data in Tokyo, despite the relatively higher use of assistive devices, short-stay respite care, and bathing, and the lower use of day care [40].

### Patterns of home services used in long-term care

Based on the descriptive data of each service used by each client, we identified frequently occurring combinations. We focused on assistive devices, day care, home helper, and visiting nurse, which were the most commonly used, and created nine combinations: day care only (n = 162, 16.5%); day care and assistive devices (n = 142, 14.4%); day care, home helper, and assistive devices (n = 130, 13.2%); home helper and assistive devices (n = 116, 11.8%); assistive devices only (n = 107, 10.9%); home helper only (n = 86, 8.7%); day care and home helper (n = 76, 7.7%); home helper, visiting nurse, and assistive devices (n = 53, 5.4%); and others (n = 111, 11.3%).

We also examined the distribution of the remaining minor services (i.e., short-stay respite care, bathing, and home rehabilitation) in each pattern (Table 2). This descriptive analysis revealed that short-stay respite care services were commonly combined with day care, while bathing were commonly combined with home helper services and assistive devices, but not with day care.

### Factors related to service use combinations: CHAID

**Figure 1:**
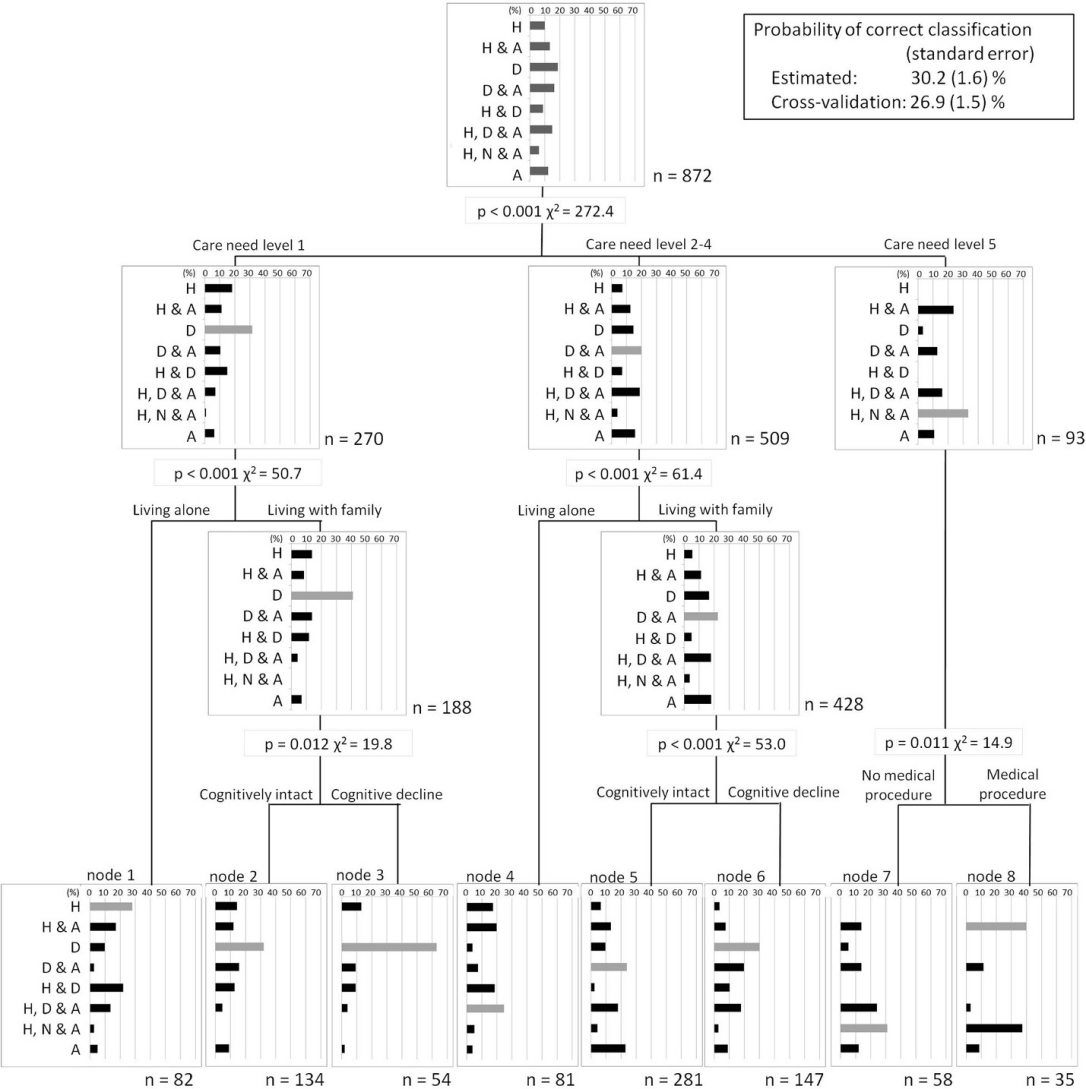
**Dendrogram for combination patterns of service utilization.** The dendrogram illustrates significant independent variables determined by chi-square value in categorizing the combinations of service uses. D: day care, H: home helper, A: assistive devices, N: visiting nurse.

To clarify the factors related to the combinations of services, which we identified from the descriptive data, we conducted the CHAID analysis. We used only eight combinations of services (the “others” category was excluded) in the analysis. The CHAID dendrogram illustrated the relative importance of significant independent variables in determining the combinations of services. The independent variables in the model were the care need level, living arrangement, cognitive function, and need for medical procedures. The characteristics of agencies or CMs were not associated with any of the eight combinations of LTCI services (Figure 1).

On the first branch of the dendrogram, the certified care need levels were divided into 3 nodes (levels 1, 2–4, and 5); the node of care need levels 2–4 and 5 included assistive devices more frequently than level 1, and the node of care need level 5 included the use of visiting nurse services more frequently than levels 1 and 2–4. On the second branch, the use of home help and day care were divided by the living arrangement; clients who lived alone used home help services more frequently, while those who lived with family used day care services more frequently. On the third branch, the use of day care and other services were divided by cognitive function; clients with cognitive decline used only day care more frequently, while cognitively intact clients also used other services, such as assistive devices.Finally, the dendrogram divided the clients into eight nodes: (1) care need level 1 and living alone (n = 82); (2) care need level 1, living with family, and cognitively intact (n = 134); (3) care need level 1, living with family, and cognitive decline (n = 54); (4) care need level 2–4 and living alone (n = 81); (5) care need level 2–4, living with family, and cognitively intact (n = 281); (6) care need level 2–4, living with family, and cognitive decline (n = 147); (7) care need level 5 and no need for medical procedures (n = 58); and (8) care need level 5 and need for medical procedure (n = 35) (Figure 1).Each bar in nodes 1–8 shows the proportion of clients using the corresponding combination of services (Figure 1). The gray bar shows the most likely combination expected for each node. When we classified the clients to the most expected category represented by the gray bar, the proportion of correctly classified clients was 30.2% with a standard error of 1.6%. The cross-validated proportion of correct classification that accounted for over-fitting in the algorithm was 26.9% with 1.5% standard error. This proportion was slightly decreased from the 30.2% found using the full sample, but much higher than the 12.5% probability associated with selecting one of the eight categories at random. This suggests that the rule used in the development of the model in this study had good predictive ability.

We obtained the same groups from the additional analysis with separate categories of day care and day care with rehabilitation; therefore, in the final model, we combined “day care” and “day care with rehabilitation”.

We observed tendencies in the services used in each node. First, assistive devices were utilized by clients with a relatively severe care need level (levels 2–5; nodes 4–8) rather than the lightest care need level (level 1; nodes 1–3).

Second, differences in service utilizations were observed by living arrangements. Most clients who lived alone in both care levels 1 (node 1) and 2–4 (node 4) used home helpers, and in some cases, day care as well. On the other hand, most clients who lived with their family (nodes 2, 3, 5, and 6) used day care. Among the clients living with family, however, cognitively intact clients (nodes 2 and 5) were more likely to rent an assistive device in addition to using day care, while almost all clients with cognitive decline (nodes 3 and 6) did not utilize the rental service.

Third, among clients with the most severe care need level (level 5), most clients who needed a medical procedure (node 8) utilized a home helper and rented an assistive device, and half of them also used visiting nurse services. On the other hand, clients who did not require medical procedures (node 7) had a different trend from those needing a procedure; some utilized day care in addition to home helper services and assistive devices.

## Discussion

In this study, we examined the use of combinations of Japanese LTCI community-based services and identified some factors associated with these combinations. To the best of our knowledge, this is the first study to report actual combinations of community-based services in Japan. Nine combinations of services were found; the CHAID techniques divided clients into eight groups by trends in the use of services.

The first important finding of this study was the identification of combined community-based services. The rates of renting assistive devices and home helper services were high (64.1% and 52.1%, respectively), and they were usually used in combination with other services (in many cases, day care) rather than alone. On the other hand, day care, which was also one of the most common services, was sometimes the only service accessed; however, in other instances, it was also used with assistive devices or home helper services. Furthermore, the results revealed that short-stay respite care was often combined with day care for the common purpose of respite for the caregiver [41]. Bathing at home was combined with home helper or visiting nurse services, but not with day care; this is logical because day care also provides bathing in the day care facility [42].

Our second finding was that clients who used community-based LTCI services were represented by eight different groups identified by care need level, cognitive function, living arrangements, and need for medical procedures. The findings that service use was associated with care need level reflecting physical disability [7–9, 11], cognitive function [18], and living arrangement [8, 10, 11, 16] were consistent with previous studies. On the other hand, we did not find associations of diagnoses and symptoms with service use, perhaps because they had smaller impacts than physical and psychological conditions that also reflected diagnoses and symptoms.

Clients with higher levels of care need were more likely to rent assistive devices. National statistics indicate that beds, bed supplements, and wheelchairs are a significant portion of assistive devices used by clients with higher care need levels [21]; most of those with lower care need levels do not need these devices; other services, such as day care, are a higher priority. Additionally, among older adults living with their family and who had either a lower care level (nodes 2 and 3) or higher care level (nodes 5 and 6), clients with cognitive decline were less likely to use assistive devices than cognitively intact clients. This result suggests that clients with cognitive decline used respite services (day care and short-stay respite care) to the limit of the family budget or the maximum benefit of the LTCI to reduce the caregiver’s burden; therefore, they could not afford to use additional services. Furthermore, this suggests a difference in services needed by clients with dementia: for instance, they might not need assistive devices because their care need levels were determined mainly based on cognitive decline and behavior associated with the decline rather than physical disability. We should take into account of the amount and types of services necessary for clients with dementia and their families in the future.

Combinations of services were related to living arrangements: older adults living alone had a home helper and those living with family used day care as the base of their care. This difference suggests that living arrangements impact service use, despite the government policy of providing appropriate services irrespective of family conditions [4].

Visiting nurse services were utilized by the most severe care need level (level 5), consistent with a previous study [2], regardless of whether medical procedures were required. In Japan, visiting nurses manage the chronic conditions of older adults and conduct necessary medical procedures [13]. Furthermore, at this care need level, implementation of medical procedures determined the use of day care: older adults who required medical procedures did not use day care, while those not requiring medical procedures used day care. This is representative of the current issue that older adults with medical procedures are not able to attend day care because the service usually has few medical professionals (only one nurse is required for day care programs). Thus, older people who require medical procedures might have limited access to respite services despite the high burden on the family. It is necessary to further investigate and offer respite services for families of clients with high medical needs.

Our third important finding was that the difference in utilization of services was not associated with the characteristics of agencies or CMs for the participants in this study; the CHAID techniques did not identify any characteristics of agencies or CMs associated with service use. Although previous studies [2, 20] pointed out the potential for bias in the selection of services or the patterns of services based on the characteristics of agencies or CMs, the effect of these factors seemed to be less than those related to the physical, psychological, and social characteristics of clients.

This study has several limitations. First, this study was conducted in only 10 agencies of the four corporations that agreed to participate in the quality evaluation and improvement project, and these agencies are located in areas around Tokyo; the selection bias might affect the results of service use and related factors. For example, there was no corporation operated by a social welfare organization (which consists of about 25% of care management agencies [43]) in this study. Further, there are some differences in the rate of use of services compared to that in the population data of Tokyo; this might indicate bias in the distribution of service agencies in the area. In addition, the agencies participating in this study used the MDS-HC as a client assessment tool; they could be highly motivated to improve their care quality. This might account for the lack of association between the patterns of services and the characteristics of agencies and CMs. In addition, the characteristics of the population and available services in Tokyo, the major metropolitan area in Japan, are different from those on a national scale.

A second limitation is that we did not investigate the amount of services or the characteristics of care service agencies selected by the CMs (e.g., if the service agencies were operated by same corporation as the care management agency). These variables might be affected by the characteristics of agencies and CMs. Future studies should examine in more detail whether the patterns of services found in this study can be applied nationwide, and re-examine the effects of characteristics of agencies and CMs on these patterns.

Despite these limitations, this study is the first to characterize combinations of community-based services use in the Japanese LTCI. The categories identified in this study can be used in care interventions in future research, which involves evaluating client outcomes (e.g., their physical or cognitive decline, hospitalization, and burden of family care givers). The possible imbalances in service use due to cognitive function, family condition, and medical procedures can be used in the continuing development of a quality care system. Finally, researchers in other countries can use the methods and results of this study to analyze how long-term care services are used and to identify the current issues in long-term care systems in each country.

## Conclusions

This study clarified combinations of community-based services and factors associated with each combination in the Japanese LTCI system. Nine categories of service use were found: (1) day care; (2) day care and assistive devices; (3) day care, home helper, and assistive devices; (4) home helper and assistive devices; (5) assistive devices; (6) home helper; (7) day care and home helper; (8) home helper, visiting nurse, and assistive devices; and (9) others. The use of services was determined by care need level, cognitive function, living arrangements, and medical procedures rather than characteristics of care management agencies and CMs. Researchers and policymakers can use these results to review the use of community-based care services and to measure outcomes of care interventions.

## Abbreviations

ADL: Activities of daily living
CHAID: Chi-squared automatic interaction detection
CM: Care manager
CPS: Cognitive performance scale
DRS: Depression rating scale
LTCI: Long-term care insurance
MDS-HC: Minimum data set-home care
SD: Standard deviation.

## References

[1] Matsuda S, Yamamoto M (2001). Long-term care insurance and integrated care for the aged in Japan. Int J Integr Care.

[2] Kashiwagi M, Tamiya N, Sato M, Yano E (2013). Factors associated with the use of home-visit nursing services covered by the long-term care insurance in rural Japan: a cross-sectional study. BMC Geriatr.

[3] Murashima S, Nagata S, Magilvy JK, Fukui S, Kayama M (2002). Home care nursing in Japan: a challenge for providing good care at home. Public Health Nurs.

[4] Tamiya N, Noguchi H, Nishi A, Reich MR, Ikegami N, Hashimoto H, Shibuya K, Kawachi I, Campbell JC (2011). Population ageing and wellbeing: lessons from Japan’s long-term care insurance policy. Lancet.

[5] Ministry of Health, Labour and Welfare (2012). [Status Report on the Long-term Care Insurance Projects].

[6] Kasahara S, Shirasawa M (2009). [Factors determining utilization of home-help service]. Koseinoshihyo.

[7] Miller B, McFall S (1991). The effect of caregiver’s burden on change in frail older persons’ use of formal helpers. J Health Soc Behav.

[8] Alkema GE, Reyes JY, Wilber KH (2006). Characteristics associated with home- and community-based service utilization for Medicare managed care consumers. Gerontologist.

[9] Li IC, Fann S, Kuo HT (2011). Predictors of the utilization of long-term care (LTC) services among residents in community-based LTC facilities in Taiwan. Arch Gerontol Geriatr.

[10] Hammar T, Rissanen P, Perala ML (2008). Home-care clients’ need for help, and use and costs of services. Eur J Ageing.

[11] Blomgren J, Martikainen P, Martelin T, Koskinen S (2008). Determinants of home-based formal help in community-dwelling older people in Finland. Eur J Ageing.

[12] Tashiro K, Sugisawa H (2010). [Factors related to adult daycare utilization among the elderly and their family caregivers]. Jpn J Gerontol.

[13] Nagata S, Taguchi A, Naruse T, Kuwahara Y, Murashima S (2010). [Actual situation and characteristics of clients judged to need home-visiting nurse services by certified care managers and comparison of users and non-users of such services]. Nihon Koshu Eisei Zasshi.

[14] de Meijer CA, Koopmanschap MA, Koolman XH, van Doorslaer EK (2009). The role of disability in explaining long-term care utilization. Med Care.

[15] Kaneko S, Ogata T, Kanekawa M (2012). [Rate of service utilization and related factors among community-dwelling older adults with cerebral stroke by care need levels]. Koseinoshihyo.

[16] de Meijer C, Koopmanschap M, D’ Uva T, van Doorslaer E (2011). Determinants of long-term care spending: age, time to death or disability?. J Health Econ.

[17] Andersen R, Newman JF (1973). Societal and individual determinants of medical care utilization in the United States. Milbank Mem Fund Q Health Soc.

[18] Bass DM, Looman WJ, Ehrlich P (1992). Predicting the volume of health and social services: integrating cognitive impairment into the modified Andersen framework. Gerontologist.

[19] Kosloski K, Montgomery RJ (1994). Investigating patterns of service use by families providing care for dependent elders. J Aging Health.

[20] Ozawa MN, Nakayama S (2005). Long-term care insurance in Japan. J Aging Soc Policy.

[21] Ministry of Health, Labour and Welfare: **[Overview of the Survey of Long-term Care Benefit Expenditures in 2012]**. [http://www.mhlw.go.jp/toukei/saikin/hw/kaigo/kyufu/12/index.html] (in Japanese)

[22] Mitsubishi Research Institute, Inc (2012). [Study of the Practices of Care Managers and Actual Situations in Human Resource Development in Care Management Agencies]. Report of Project on Health and Welfare for the Elderly.

[23] Suda Y, Kodama H (2010). Use Of Long-Term Care Services And Family.

[24] Hirano T, Okuda Y, Sasagawa O, Huzita K, Nakajima T (2007). [Analysis of service packages in long-term care insurance among older adults with dementia in an urban area]. Koseinoshihyo.

[25] Choi S, Morrow-Howell N, Proctor E (2006). Configuration of services used by depressed older adults. Aging Ment Health.

[26] Kendig H, Mealing N, Carr R, Lujic S, Byles J, Jorm L (2012). Assessing patterns of home and community care service use and client profiles in Australia: a cluster analysis approach using linked data. Health Soc Care Commun.

[27] Hong SI (2010). Understanding patterns of service utilization among informal caregivers of community older adults. Gerontologist.

[28] Diwan S, Berger C, Manns EK (1997). Composition of the home care service package: predictors of type, volume, and mix of services provided to poor and frail older people. Gerontologist.

[29] Hirdes JP, Fries BE, Morris JN, Ikegami N, Zimmerman D, Dalby DM, Aliaga P, Hammer S, Jones R (2004). Home care quality indicators (HCQIs) based on the MDS-HC. Gerontologist.

[30] Morris JN, Fries BE, Steel K, Ikegami N, Bernabei R, Carpenter GI, Gilgen R, Hirdes JP, Topinkova E (1997). Comprehensive clinical assessment in community setting: applicability of the MDS-HC. J Am Geriatr Soc.

[31] Ishibashi T, Ikegami N (2010). Should the provision of home help services be contained?: validation of the new preventive care policy in Japan. BMC Health Serv Res.

[32] Campbell JC, Ikegami N (2000). Long-term care insurance comes to Japan. Health Aff.

[33] Morris JN, Fries BE, Morris SA (1999). Scaling ADLs within the MDS. J Gerontol.

[34] Fries BE, Simon SE, Morris JN, Flodstrom C, Bookstein FL (2001). Pain in U.S. nursing homes: validating a pain scale for the minimum data set. Gerontologist.

[35] Hartmaier SL, Sloane PD, Guess HA, Koch GG, Mitchell CM, Phillips CD (1995). Validation of the minimum data set cognitive performance scale: agreement with the Mini-Mental State Examination. J Gerontol A Biol Sci Med Sci.

[36] Burrows AB, Morris JN, Simon SE, Hirdes JP, Phillips C (2000). Development of a Minimum Data Set-based depression rating scale for use in nursing homes. Age Ageing.

[37] Kass GV (1980). An exploratory technique for investigating large quantities of categorical data. J R Stat Soc: Ser C: Appl Stat.

[38] Naruse T, Nagata S, Taguchi A, Kuwahara Y, Murashima S (2012). Characteristics of family caregivers with sleep dissatisfaction in Japan: identification using CHAID dendrograms. Biosci Trends.

[39] Naruse T, Nagata S, Taguchi A, Murashima S (2011). Classification tree model identifies home-based service needs of Japanese long-term care insurance consumers. Public Health Nurs.

[40] Ministry of Health, Labour and Welfare: **[Survey of Long-term Care Benefit Expenditures]**. [http://www.mhlw.go.jp/toukei/list/45-1.html] (in Japanese)

[41] Lopez-Hartmann M, Wens J, Verhoeven V, Remmen R (2012). The effect of caregiver support interventions for informal caregivers of community-dwelling frail elderly: a systematic review. Int J Integr Care.

[42] Traphagan JW (2004). Culture and long-term care: the bath as social service in Japan. Care Manag J.

[43] Ministry of Health, Labour and Welfare: **[Survey of Institutions and Establishments for Long-term Care 2012]**. [http://www.mhlw.go.jp/toukei/saikin/hw/kaigo/service12/index.html] (in Japanese)

[CR44] The pre-publication history for this paper can be accessed here: http://www.biomedcentral.com/1472-6963/14/382/prepub

